# Vaccine-induced antibodies to herpes simplex virus glycoprotein D epitopes involved in virus entry and cell-to-cell spread correlate with protection against genital disease in guinea pigs

**DOI:** 10.1371/journal.ppat.1007095

**Published:** 2018-05-23

**Authors:** Lauren M. Hook, Tina M. Cairns, Sita Awasthi, Benjamin D. Brooks, Noah T. Ditto, Roselyn J. Eisenberg, Gary H. Cohen, Harvey M. Friedman

**Affiliations:** 1 Infectious Disease Division, Department of Medicine, Perelman School of Medicine, University of Pennsylvania, Philadelphia, Pennsylvania, United States of America; 2 Department of Microbiology, School of Dental Medicine, University of Pennsylvania, Philadelphia, Pennsylvania, United States of America; 3 Carterra, Inc., Salt Lake City, Utah, United States of America; 4 Department of Pathobiology, School of Veterinary Medicine, University of Pennsylvania, Philadelphia, Pennsylvania, United States of America; Louisiana State University Health Sciences Center, UNITED STATES

## Abstract

Herpes simplex virus type 2 (HSV-2) glycoprotein D (gD2) subunit antigen is included in many preclinical candidate vaccines. The rationale for including gD2 is to produce antibodies that block crucial gD2 epitopes involved in virus entry and cell-to-cell spread. HSV-2 gD2 was the only antigen in the Herpevac Trial for Women that protected against HSV-1 genital infection but not HSV-2. In that trial, a correlation was detected between gD2 ELISA titers and protection against HSV-1, supporting the importance of antibodies. A possible explanation for the lack of protection against HSV-2 was that HSV-2 neutralization titers were low, four-fold lower than to HSV-1. Here, we evaluated neutralization titers and epitope-specific antibody responses to crucial gD2 epitopes involved in virus entry and cell-to-cell spread as correlates of immune protection against genital lesions in immunized guinea pigs. We detected a strong correlation between neutralizing antibodies and protection against genital disease. We used a high throughput biosensor competition assay to measure epitope-specific responses to seven crucial gD2 linear and conformational epitopes involved in virus entry and spread. Some animals produced antibodies to most crucial epitopes while others produced antibodies to few. The number of epitopes recognized by guinea pig immune serum correlated with protection against genital lesions. We confirmed the importance of antibodies to each crucial epitope using monoclonal antibody passive transfer that improved survival and reduced genital disease in mice after HSV-2 genital challenge. We re-evaluated our prior study of epitope-specific antibody responses in women in the Herpevac Trial. Humans produced antibodies that blocked significantly fewer crucial gD2 epitopes than guinea pigs, and antibody responses in humans to some linear epitopes were virtually absent. Neutralizing antibody titers and epitope-specific antibody responses are important immune parameters to evaluate in future Phase I/II prophylactic human vaccine trials that contain gD2 antigen.

## Author summary

Herpes simplex virus type 2 (HSV-2) glycoprotein D (gD2) mediates virus entry and cell-to-cell spread. We hypothesized that an effective gD2 vaccine needs to block these activities. Neutralizing titers, which assess blocking virus entry, correlated with protection against HSV-2 genital lesions in gD2-immunized guinea pigs. We used a high throughput biosensor assay to evaluate whether gD2-immunized guinea pigs produce antibodies that block the activities of gD2 epitopes involved in virus entry and cell-to-cell spread. We detected a strong correlation between the number of epitopes blocked by immune serum and protection against genital lesions. Passive transfer of monoclonal antibodies in mice infected intravaginally with HSV-2 confirmed the *in vivo* importance of antibodies to these epitopes. We re-evaluated our prior report of epitope-specific antibody responses in the gD2 Herpevac Trial, a trial that failed to protect subjects against HSV-2 genital infection. We detected antibody responses to significantly fewer epitopes in immunized humans than guinea pigs. We conclude that neutralizing antibodies and antibodies to multiple gD2 epitopes involved in virus entry and cell-to-cell spread represent important immune correlates of protection. Future human trials that contain gD2 as an immunogen need to assess these responses and correct deficiencies prior to embarking on large trials.

## Introduction

Nearly one half-billion people worldwide are infected with herpes simplex virus type 2 (HSV-2) and another one-quarter billion have genital infection caused by herpes simplex virus type 1 (HSV-1) [1, 2]. The disease manifestations vary from asymptomatic genital infection to severe, recurrent ulcerative genital disease [3–5]. Genital herpes poses major risks to newborns delivered through an infected birth canal with an estimated 14,000 annual cases globally and a mortality of 60% if untreated [2, 6]. HSV-2 genital infection carries a three-fold increased risk for acquisition and transmission of HIV [7].

A vaccine to prevent genital herpes is urgently needed, yet none has been approved despite several clinical trials evaluating the efficacy of potential vaccine candidates [8–10]. The Herpevac Trial for Women was the most recent large prophylactic genital herpes human vaccine trial that involved immunizing HSV-1 and HSV-2 doubly seronegative women with gD2 subunit antigen [10]. The vaccine protected against HSV-1 genital infection, but not HSV-2. Higher ELISA antibody titers were associated with reduced acquisition of HSV-1, highlighting the importance of antibodies for protection against genital herpes [10]. Vaccine-induced antibodies correlate with protection against other sexually transmitted infections, including human papilloma virus (HPV) and hepatitis B virus (HBV) [11]. Therefore, our strategy has focused on the generation of robust antibody responses for a prophylactic HSV-2 vaccine.

HSV-2 prophylactic vaccine antibody responses have generally targeted virus entry molecules [8–10]. Glycoprotein D is one of four glycoproteins essential for virus entry into cells. The other glycoproteins are gB, gH, and gL [12, 13]. Glycoprotein B, along with gC facilitates the initial attachment of virus to host cells by interaction with heparan sulfate proteoglycans. Glycoprotein D then binds to cell receptors nectin-1, herpesvirus entry mediator (HVEM), or 3-*O*-sulfotransferase heparan sulfate an event that induces conformational changes in gD, allowing it to activate the gH/gL heterodimer complex [14, 15]. Activation of gH/gL transitions gB into a fusogenic state that facilitates fusion of the virus and host cell membranes and subsequent entry of the virus capsid into the cell [16–19]. Glycoprotein D is also required for efficient spread of virus from one cell to another [20, 21]. Domains on gD involved in cell-to-cell spread are thought to differ from those involved in virus entry [22, 23]. Identifying the location on gD of these important functional domains was aided by solving the atomic structure of gD [24].

We have assembled a large panel of anti-gD MAbs that we used to assess antibody responses to specific gD2 linear and conformational epitopes elicited by the gD2 vaccine in a subset of 29 human volunteers in the Herpevac trial [25]. Individuals that produced antibodies to the greatest number of neutralizing gD2 epitopes, as measured by antibody competition assays, had the highest neutralizing antibody titers, suggesting that a diverse antibody response correlates with neutralizing activity [25]. The Herpevac trial did not protect against HSV-2 genital infection; therefore, we were not able to assess whether antibody responses to specific gD2 epitopes correlate with protection against genital herpes.

We recently evaluated a trivalent vaccine consisting of HSV-2 glycoproteins C, D and E (gC2, gD2, and gE2) aimed at improving antibody responses by blocking virus entry mediated by gD2 and immune evasion from antibody and complement mediated by gE2 and gC2, respectively [26]. The trivalent vaccine was 97% efficacious in reducing the number of days with genital lesions compared with mock-immunized animals [26]. Some guinea pigs received gD2 alone as comparison for vaccine efficacy with the trivalent vaccine. The gD2 alone group was 80% efficacious in reducing the number of days with genital lesions compared with mock-immunized animals, and 33% efficacious in totally preventing genital lesions. The observation that some animals in the gD2 alone group were fully protected while others had lesions on one or more days provided an opportunity to correlate responses to specific gD2 epitopes with the level of protection against disease.

Passive antibody transfer studies have been used to evaluate protection provided by antibodies against HSV-2 intravaginal infection [27, 28]. We used the murine genital infection model to demonstrate that passive transfer of individual gD type common and type specific MAbs that bind to crucial gD2 epitopes required for virus entry or cell-to-cell spread *in vitro* provide protection against intravaginal challenge *in vivo*. We used the guinea pig model of genital infection to correlate antibodies produced to specific epitopes with protection against genital lesions after intravaginal challenge in gD2-immunized guinea pigs. We noted that neutralizing and gD2 ELISA antibody titers correlated with protection as did producing antibodies to multiple crucial gD2 epitopes. We used this information to re-evaluate the gD2 immune responses of humans immunized with the HSV-2 gD2 vaccine in the Herpevac Trial. Our results demonstrate that antibody responses to some crucial gD2 epitopes that are protective in animal models were absent in the human vaccine trials.

## Results

### Guinea pig immunization studies

Twenty-five animals immunized with gD2 subunit antigen with CpG and alum as adjuvants were evaluated in two prophylactic vaccine studies [26]. All 25 animals survived intravaginal challenge; however, 16/25 animals developed genital lesions [26]. We performed a secondary analysis of correlates of protection against genital lesions based on the previously collected neutralizing antibody and genital lesions data. In addition, we generated new results that assessed endpoint ELISA antibody titers, monoclonal antibody (MAb) passive transfer, and high throughput biosensor assays. We restricted our analysis to protection against genital lesions since too few animals, only 4 of 25 (16%), were totally protected against any evidence of infection, defined as no genital lesions and no subclinical shedding of HSV-2 DNA in genital secretions [26].

### Neutralizing and ELISA antibody responses in gD2-immunized guinea pigs correlate with protection against genital lesions

**Fig 1:**
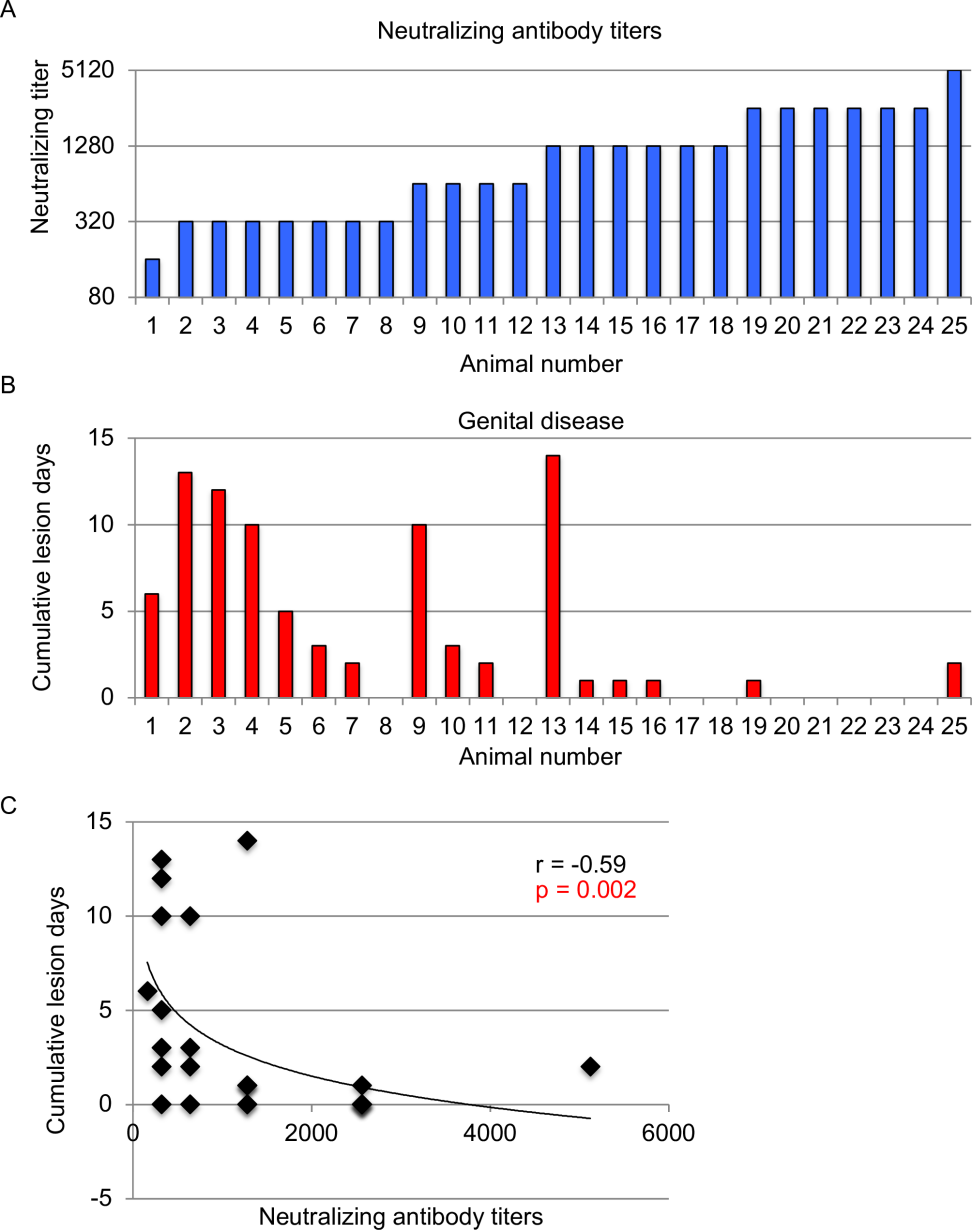
Neutralizing antibody responses correlate with protection against HSV-2 genital lesions. (A) Serum neutralizing antibody titers are plotted from guinea pigs immunized with gD2. Serum was obtained after the third immunization. Animals are ranked from lowest to highest neutralizing antibody titer. (B) Cumulative lesion days per animal were monitored for 60 days post infection. Animals are ordered as in A. (C) The correlation between cumulative lesion days and serum neutralizing titers. P and rho (r) values were calculated by Spearman's correlation.

The previously reported serum neutralizing antibody titers without complement are aligned from lowest to highest neutralizing antibody titers and ranged from 1:160 to 1:5120 in these outbred animals (geometric mean titer 1:893) (Fig 1A) [26]. We listed the animals in the same order and plotted cumulative days with genital lesions over a period of 60 days post-challenge. Nine animals had no genital lesions, while 16 had genital lesions on one to 14 days (Fig 1B). We performed regression analysis and found that higher neutralizing titers correlated significantly with fewer lesions days (Fig 1C). We conclude that neutralizing antibodies are important for protection of these gD2-immunized animals; however, other immune responses associated with gD2 immunization also likely contribute to protection since the correlation was strong, but not perfect. For example, animals #19 and 25 with neutralizing titers of 1:2560 and 1:5120, respectively developed genital lesions.

**Fig 2:**
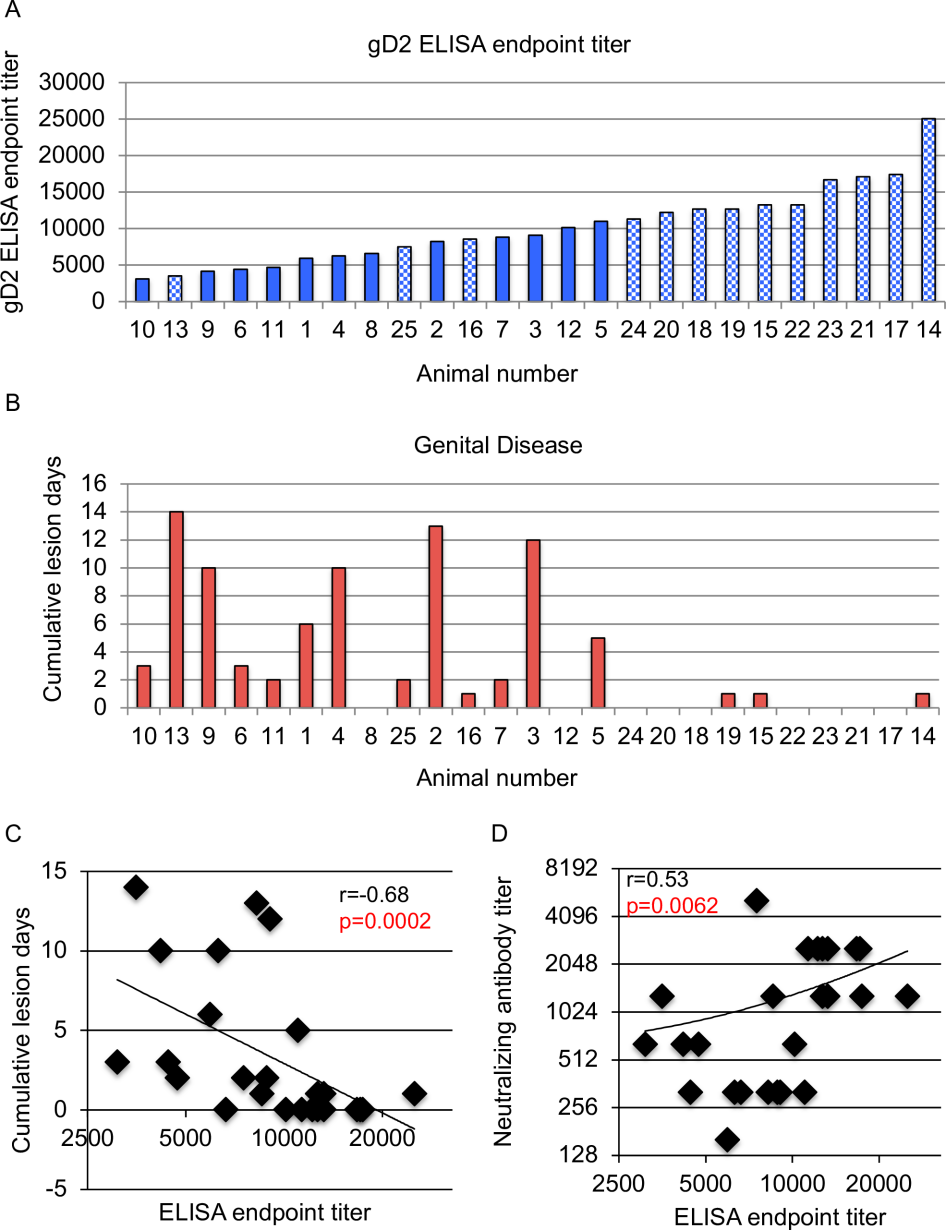
ELISA antibody responses correlate with protection from HSV-2 genital lesions. (A) ELISA endpoint titers are plotted from serum obtained after the third immunization. Animals are ranked from lowest to highest ELISA titer. Animal numbers are the same as assigned in Fig 1. The hatched bars represent animals with neutralizing titers ≥1:1280. (B) Cumulative lesion days per animal monitored for 60 days post infection. Animals are ordered as in A. (C) The correlation between cumulative lesion days and serum ELISA titers. (D) The correlation between neutralizing antibody titers and gD2 ELISA endpoint titers. P and rho (r) values in C and D were calculated by Spearman's correlation.

We performed a similar analysis comparing gD2 ELISA endpoint titers with genital lesions, since a correlation between ELISA titers and protection against genital HSV-1 infection was noted in the Herpevac Trial for Women [10, 29]. Each animal was assigned the same number as in Fig 1, although the order of the animals changed based on lowest to highest ELISA titers that ranged from 1:3,000 to 1:25,000, with a geometric mean titer of 1:8922 (Fig 2A). We noted a significant correlation between higher ELISA titers and fewer cumulative lesion days (Fig 2B and 2C), which is consistent with the HSV-1 results in the Herpevac Trial for Women [10, 29].

Neutralizing antibodies reflect a mechanism of protection, such as blocking virus entry, while ELISA titers indicate antibody binding but do not define a specific mechanism by which the antibodies protect. We analyzed whether neutralizing antibody and ELISA titers correlate with one another (Fig 2D). The relatively strong correlation between these two assays for measuring antibody responses indicates that neutralizing antibodies are an important mechanism for protection. Since the correlation was not perfect (that is, it was <1.0), it suggests that other immune mechanisms also contribute to overall protection.

### Antibody responses in gD2-immunized guinea pigs to specific epitopes that mediate crucial gD2 functions

**Fig 3:**
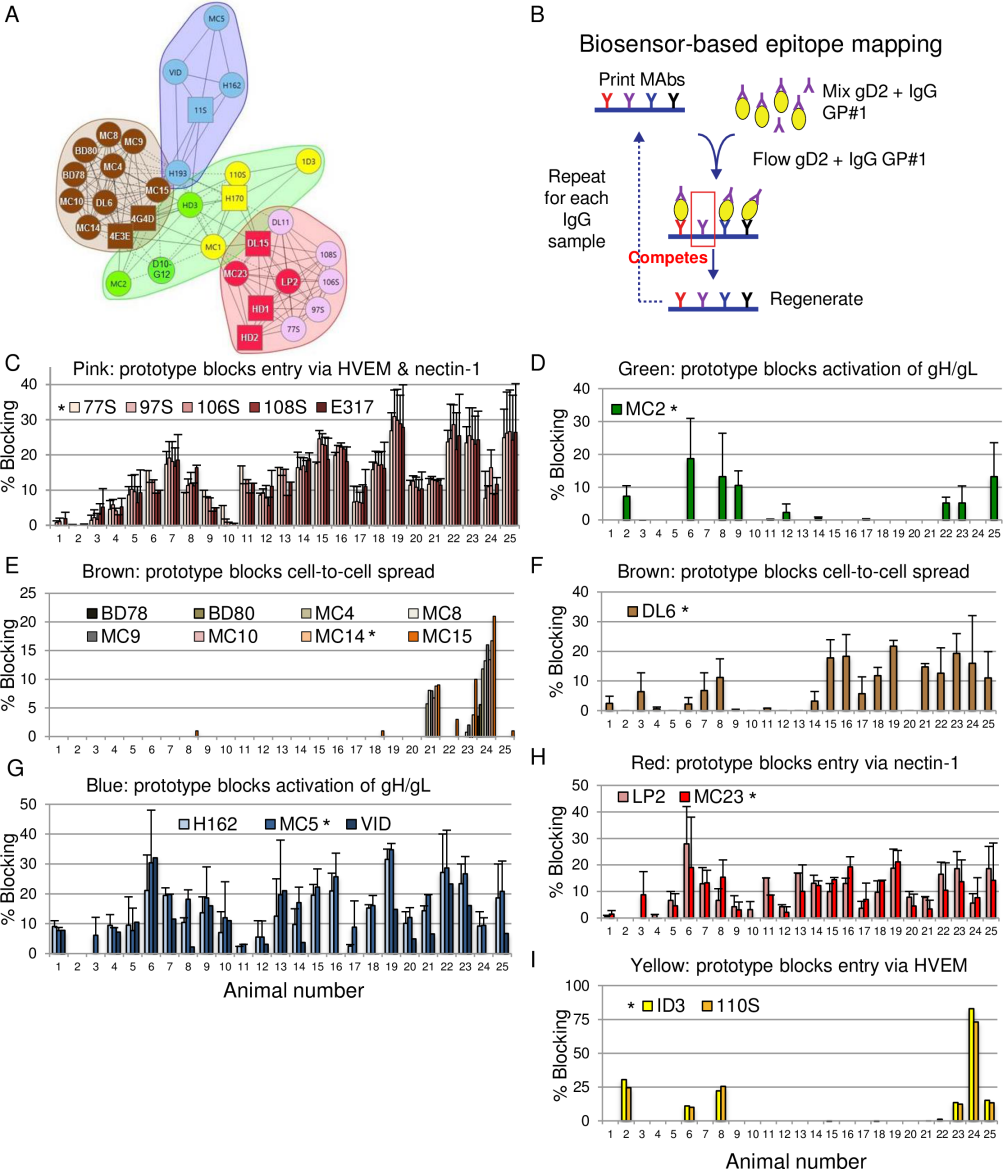
Biosensor-based epitope binning. (A) Properties of gD MAbs. MAbs that compete for binding are grouped into communities. Each community is sorted by color. The communities shown in A are reproduced with modifications from our prior publication by Cairns et al [30]. (B) Schematic of the biosensor based MAb competition assay. IgG from guinea pig #1 is incubated with gD2(285t) and flowed over the panel of MAbs printed on the biosensor chip. The gD2 fails to bind to one or more of the printed MAbs if IgG in the guinea pig serum competes with the printed MAb. The gD2 is stripped off the printed MAbs and the process is repeated with IgG from each of the other guinea pigs until all 25 have been evaluated. (C-I) Percent blocking of gD2(285t) binding to printed MAbs that recognize epitopes involved in receptor binding, activation of gH/gL and cell-to-cell spread. Each animal is assigned the same number as in Figs 1 and 2. Each guinea pig IgG was tested twice, except animal #11 was evaluated only once. Each prototype MAb was evaluated in both runs, except MAbs 1D3 and MC14 were tested once. Thus, 25 animals are in each group except that 24 animals are in the 1D3 and MC14 groups. Five MAbs were evaluated from the pink community, nine from the brown, including DL6 that is shown separately from the other brown community MAbs, three from blue, 2 from yellow, 1 from green and 2 from red. The IgG was considered positive for blocking if any blocking (≥1%) was detected. * indicates prototype MAbs within each community. Error bars represent SEM.

We used a high throughput biosensor-based MAb competition assay to assess the production of epitope-specific antibodies in immunized guinea pigs. The MAbs selected for evaluation were based on their assignment to competition-based communities and subcommunities (Fig 3A) [30]. We assessed whether antibodies are produced by immunization to specific gD2 epitopes using the approach shown schematically in Fig 3B. First, gD MAbs from the different communities were printed on the biosensor chip. Purified gD2(285t) was pre-incubated with IgG isolated from individual immunized guinea pigs and each gD2-IgG mix was flowed separately over the printed MAbs on the chip. The rationale for using gD2(285t) rather than gD2(306t) is explained in Methods.

We evaluated whether pre-incubation of gD2(285t) with IgG from individual guinea pigs blocked gD2(285t) binding to the printed MAbs. Blocking gD2(285t) binding to the printed MAb indicates that the guinea pig IgG contains antibodies that compete for binding with the printed MAb (Fig 3C–3I). Guinea pigs were assigned the same numbers as in Figs 1 and 2. Twenty-three of 25 guinea pigs produced antibodies that competed with the prototype MAb 77S in the pink community. 77S was used as the prototype MAb rather than DL11 because DL11 binding activity tends to be lost when the chip is repeatedly regenerated during antibody blocking studies. Only guinea pigs #1 and #2 failed to produce antibodies that blocked gD2 binding to prototype MAb 77S (Fig 3C). Twenty-four of 25 immunized animals produced antibodies to epitopes recognized by the blue prototype MAb MC5, with only guinea pig #2 failing to block (Fig 3G). Twenty-two of 25 guinea pigs produced antibodies that competed with the red prototype MAb MC23, with animals #2, #4 and #10 failing to block (Fig 3H). Eighteen of 25 animals produced antibodies that blocked the brown prototype MAb DL6 (Fig 3F), while many fewer animals (7/24) produced antibodies to epitopes recognized by the yellow prototype MAb 1D3 (Fig 3I), green prototype MAb MC2 (8/25) (Fig 3D) or brown prototype MAb MC14 (3/24) (Fig 3E). For most communities, we evaluated two or more MAbs and obtained highly concordant results regarding the percent blocking and whether guinea pig IgG blocked gD2 binding to the MAbs in the community. The one exception was in the brown community where DL6 (Fig 3F) had different performance characteristics than the other MAbs in that community (Fig 3E), supporting prior mapping studies that placed the DL6 epitope in group IIb, while the other brown community MAbs were grouped as IIa [25, 31]. We conclude that some crucial gD2 epitopes, such as those recognized by MAbs in the pink, blue and red communities are highly immunogenic in almost all immunized animals, while others, such as those recognized by the green, yellow and brown communities are considerably less immunogenic.

### Passive antibody transfer studies

#### Defining the properties of the non-neutralizing prototype MAbs in blocking cell-to-cell spread *in vitro*

**Fig 4:**
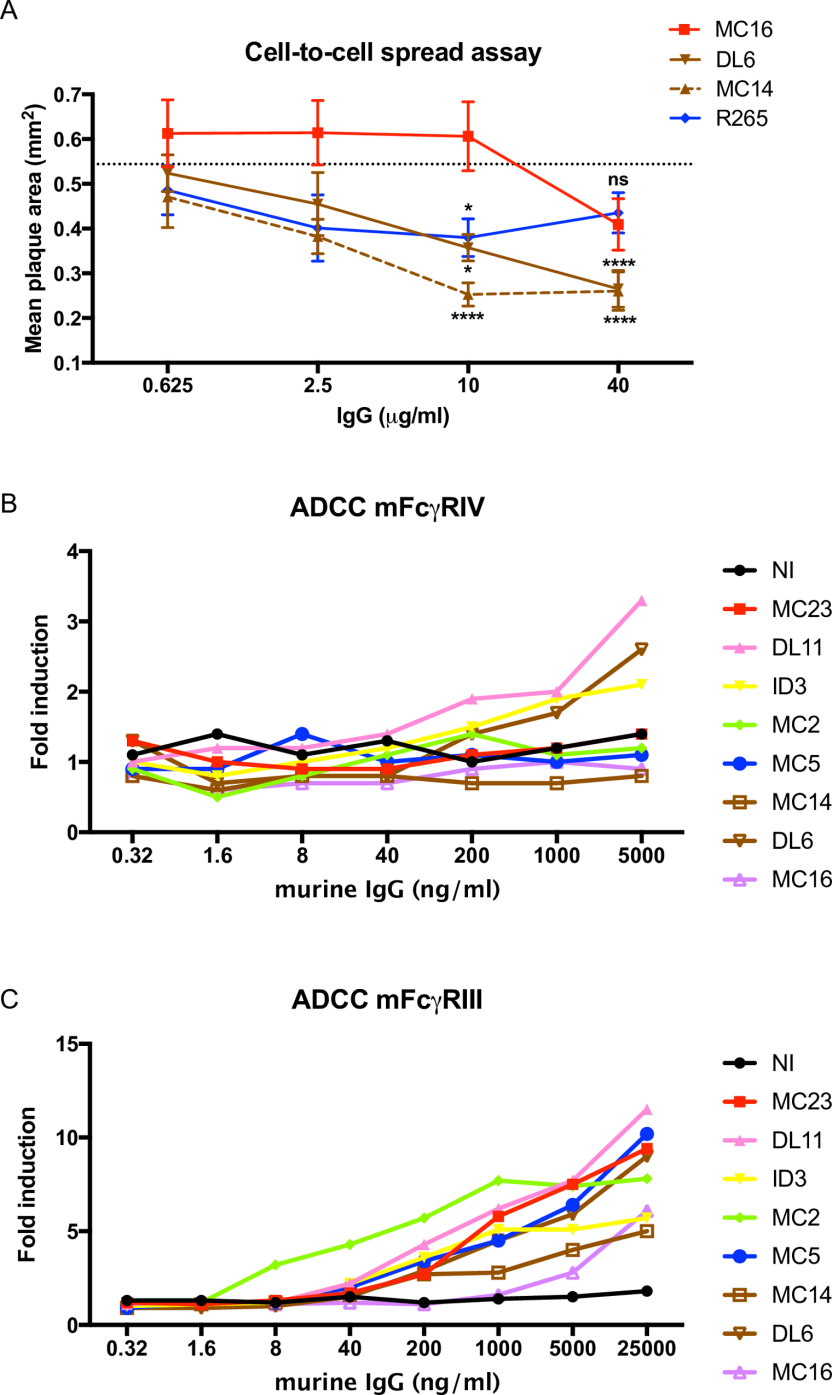
Blocking cell-to-cell spread and binding to murine FcγRIII and FcγRIV receptors by prototype MAbs. (A) MAbs MC16, DL6 and MC14 were added to HSV-2-infected HaCaT cells and plaque size was measured after 48 hours. R265 represents anti-gE2 polyclonal rabbit IgG used as a positive control. The dotted line represents plaque size in wells that contained media with no added antibodies. P values were determined by comparing plaque size in wells with added antibodies with the no-added antibody control wells. Statistics were calculated by Mann-Whitney; * p<0.05; **** p<0.0001; ns, not significant. (B-C) Binding of MAbs to murine IgG FcγRIV or FcγRIII that mediate ADCC. Endpoint titers were calculated based on the lowest MAb dilution that produced luminescence readings 2-fold higher than the no antibody control. Note that the Y-axis scales are different for B and C.

We previously reported the neutralizing endpoint titers of the prototype MAbs in the red, pink, yellow, blue and green communities and noted that MAbs DL6 and MC14 in the brown community are non-neutralizing [30]. An important function of gD2 is to mediate virus spread from cell-to-cell [20, 21]. We evaluated whether the non-neutralizing brown prototype MAbs DL6 and MC14 block cell-to-cell spread. We assessed anti-gD2 MAb MC16 as another unknown. This MAb is non-neutralizing and does not block cell-to-cell spread and has not been placed within any of the defined gD2 communities or subcommunities [30, 31]. Anti-gE2 polyclonal rabbit IgG, R265 is a non-neutralizing antibody that blocks cell-to-cell spread and was used as a positive control, while media with no antibody added was used as a negative control [32]. MC14, DL6 and the positive control R265 each significantly reduced plaque size in HSV-2 infected HaCaT cells, while MC16 had no effect (Fig 4A). The endpoint titer for MC14 was 2.5 μg/ml and for DL6 was 10 μg/ml, suggesting that MC14 is more potent at blocking cell-to-cell spread than DL6, while MC16 has no activity.

#### Defining the properties of the prototype MAbs for each community or subcommunity in mediating antibody-dependent cellular cytotoxicity (ADCC) *in vitro*

ELISA titers, neutralization in the absence of complement and blocking cell-to-cell spread measure activities mediated by the IgG Fab domain; however, HSV antibodies also mediate important functions by their Fc domains [33, 34]. Therefore, we analyzed the ADCC endpoint titers of the prototype MAbs to provide additional information on the protective properties of these MAbs. As expected, the IgG2a prototype MAbs, DL11, 1D3 and DL6 bound to the high affinity murine IgG FcγRIV receptor, while none of the IgG1 isotype MAbs or nonimmune murine IgG bound [35]. The endpoint titer for DL11 and 1D3 was 1.0 μg/ml, which was lower than DL6 at 5.0 μg/ml (Fig 4B and Table 1). The relative luminescence units (RLU) for these MAbs at the highest concentration tested ranged from 16- to 25-fold above background levels. We also measured binding of the MAbs to the low affinity murine IgG FcγRIII receptor that binds both murine IgG1 and IgG2a isotypes [35]. Each prototype MAb and the control MAb MC16 bound to the murine FcγRIII receptor with endpoint titers ranging from 8 ng/ml to 5 μg/ml (Fig 4C and Table 1). The RLU of the MAbs at the highest concentration tested ranged from 61- to 138-fold above background levels. The results indicate that the Fc domain of each prototype MAb and the control MAb are capable of binding to one or more murine FcγR, and thus can potentially mediate ADCC.

**Table 1:** Properties of MAbs used for passive transfer in mice.

Community; and prototype	Amino acids bound* [30]	Group; and linear (L) or conformational (C) epitope [31, 63–65]	Functions blocked *in vitro* [12, 37]	Avidity (off rate)^#^ [30]	Iso-type	Neutralizing endpoint titer μg/ml [30, 31]	Blocking cell-to-cell spread end-point titer μg/ml^§^	ADCC FcγRIII end-point titer μg/ml^⌘^	ADCC FcγRIV end-point titer μg/ml^¶^
Red MC23	213, 216	1a C	Entry via nectin-1	8.10E-04	IgG1	0.26	NA	0.20	No ADCC
Pink DL11	38, 132, 140, 222–224	1b C	Entry via nectin-1 & HVEM	1.0E-05	IgG2a	0.31	NA	0.04	1.0
Yellow 1D3	10–20	VIIa L	Entry via HVEM	5.45E-05	IgG2a	6.2	NA	0.04	5.0
Blue MC5	75–79	XVI C	Second function of gD: proposed activation of gH/gL	1.0E- 05	IgG1	6.2	NA	0.04	No ADCC
Green MC2	246	XVIb C	Second function of gD: proposed activation of gH/gL	2.90E-04	IgG1	0.78	NA	0.008	No ADCC
Brown DL6	272–279	IIb L	Cell-to-cell spread	4.0E-04	IgG2a	Non-neutral-izing	10	0.20	5.0
Brown MC14	262–272	IIa L	Cell-to-cell spread	6.71E-04	IgG1	Non-neutral-izing	2.5	0.20	No ADCC
None MC16	235–245	IIa L	None known	ND	IgG1	Non-neutral-izing	Does not block spread	5.0	No ADCC

* Residues bound uses a numbering system that assigns the lysine that is the first amino acid after the 26-amino acid signal sequence as amino acid #1. NA: not applicable; ND, not determined because MC16 binds poorly to gD2 on the biosensor chip;

^#^ Data derived from Table 2 the gD(285t) column in reference [30];

^§^ Data derived from Fig 4A;

^⌘^ Data derived from Fig 4C;

^¶^ Data derived from Fig 4B

#### Passive transfer of gD2 prototype MAbs in mice to assess whether antibodies to epitopes that are associated with crucial gD2 functions *in vitro* are protective *in vivo*

We evaluated the gD2 prototype MAbs from each community or subcommunity in antibody passive transfer studies in mice. The purpose was to assess whether MAbs that block crucial gD2 functions *in vitro* provide protection against lethal infection *in vivo*. As controls, we included non-immune murine IgG and MAb MC16 that binds to gD2 but does not block any known gD2 function [31]. A summary of the prototype MAbs from each community and subcommunity that were used for passive transfer is provided in Table 1. The endpoint titers of the cell-to-cell spread and ADCC assays are described in Fig 4A–4C. Three of the seven prototype MAbs recognize crucial gD2 epitopes and block receptor binding to HVEM (1D3), nectin-1 (MC23) or both receptors (DL11) and two are proposed to block gD2 activation of gH2/gL2 (MC2 and MC5), an essential complex for virus entry. The prototype MAbs that block receptor binding or the interaction between gD2 and gH2/gL2 are all neutralizing (Table 1) [30]. Two MAbs, DL6 and MC14 inhibit cell-to-cell spread (Table 1 and Fig 4) [25]. Each of the three IgG2a MAbs, DL11, 1D3 and DL6 bind to the high affinity murine FcγRIV receptor that is present on monocytes, macrophages and neutrophils [35]. All seven prototype MAbs and to a lesser extent, MAb MC16 bind to the low affinity murine FcγRIII receptor that is present on all myeloid cells and NK cells [35, 36]. The avidity (dissociation rate) of each prototype MAb ranged from higher avidity (smaller number indicating slower off rate) for DL11and MC5, intermediate for 1D3, to lower avidity (larger number indicating faster off rate) for MC2 (Table 1) [30].

**Fig 5:**
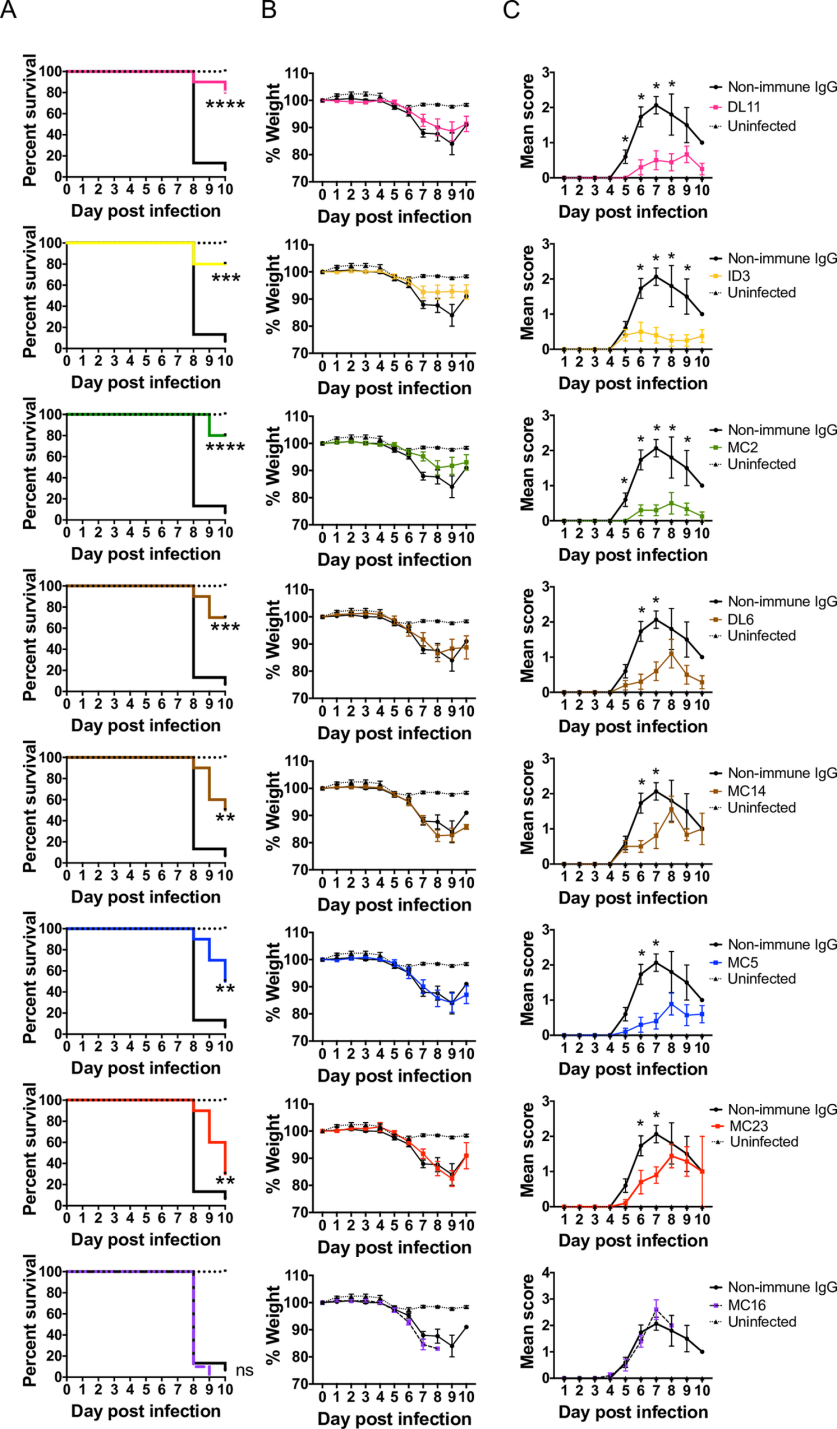
Antibody passive transfer of prototype MAbs in mice. BALB/c mice were passively immunized IP and infected the next day intravaginally with 5x10^3^ PFU HSV-2 strain MS. Animals were observed for 10 days for survival (A), weight loss (B) and genital disease (C). Weights recorded are for surviving animals, which explains the increase in average weights observed in many groups towards the end of the experiment as euthanized animals drop out. Sample size: n = 15 for non-immune IgG; n = 10 for each prototype MAb; and n = 10 for uninfected animals. The Mantel-Cox method was used to calculate P values for survival curves. Each prototype MAb provided significantly better protection than non-immune IgG (P values noted on Fig 5A), and each prototype MAb provided significantly better protection than MC16 (p<0.001). Protection provided by DL11 or MC2 (both protected 80% of mice) was significantly better than MC23 (protected 30% of mice) (p = 0.036). No significant differences were detected for weight loss in any group. Significant differences between prototype MAbs and non-immune IgG for genital disease scores are noted in Fig 6C. Student’s t-tests were used to compare MAbs with nonimmune IgG for weight loss and disease scores. * p<0.05; ** p<0.01; *** p<0.001; **** p<0.0001.

BALB/c mice were injected intraperitoneally with each gD2 MAb or nonimmune murine IgG and 1 day later infected intravaginally with 5X10^3^ PFU of HSV-2 strain MS (500 LD_50_), a highly lethal inoculum in naïve mice. The primary endpoint was survival, while secondary endpoints were protection against weight loss and genital disease. Compared with nonimmune murine IgG, passive transfer of gD2 MAbs from each community or subcommunity significantly protected mice against death, ranging from 80% protection with a member of the pink (DL11), yellow (1D3) and green (MC2) MAbs to 30% protection with red (MC23). The community control MAb MC16 failed to protect mice (Fig 5A). Therefore, each MAb to an epitope crucial for gD2 function *in vitro* was protective to varying extents *in vivo*. Weight loss occurred in all groups and was not significantly different comparing prototype MAbs and nonimmune IgG for any group (Fig 5B). Genital disease developed in all groups, and the disease scores followed a similar pattern as survival with each prototype MAb providing significant protection (Fig 5C). Pink (DL11), yellow (1D3) and green (MC2) MAbs were most protective against genital disease, while brown (DL6 and MC14) and blue (MC5) MAbs provided intermediate protection, and the red (MC23) MAb offered only limited protection. The control MAb (MC16) provided no protection. Therefore, a single MAb was sufficient to improve survival and reduce the severity of genital disease, but did not prevent weight loss.

### Correlation between the number of gD2 epitopes blocked and protection against genital lesions in guinea pigs

**Fig 6:**
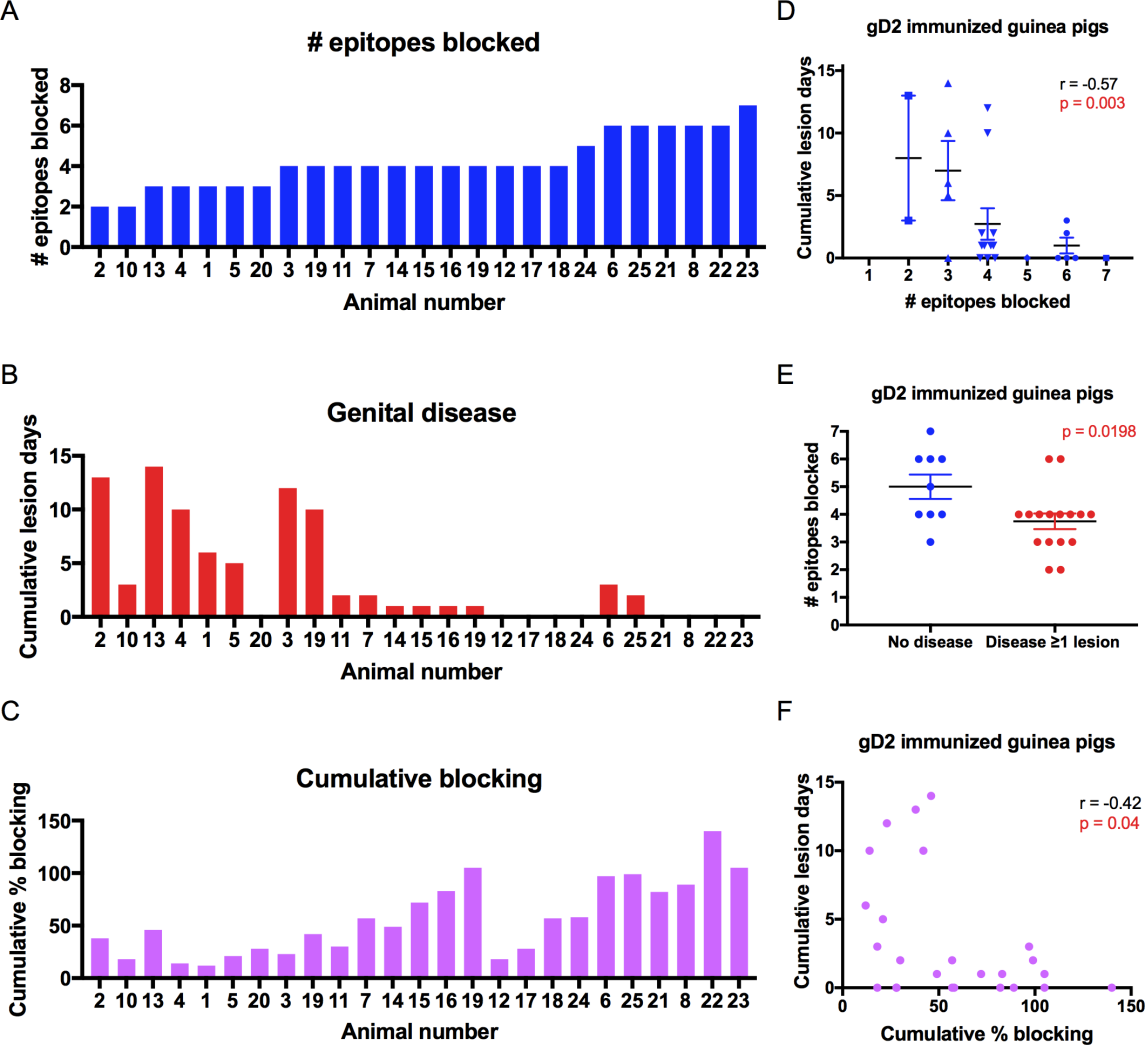
Correlation between the number of epitopes blocked and genital lesions. (A) Number of epitopes blocked by IgG from each guinea. Animals are ranked from lowest to highest number of epitopes blocked. (B) Cumulative lesion days per animal monitored for 60 days post infection. Animals are ordered as in A. (C) Cumulative percent epitope blocking by IgG from each guinea pig. (D) Correlation between the cumulative lesion days and number of epitopes blocked. The P value was determined by Spearman's correlation. (E) Comparing animals with no lesion days with animals that had ≥1 lesion day. The P value was determined by the Students t-test. Horizontal bars in D and E represent means and SEM. (F) Correlation between the cumulative lesion days and the cumulative percent epitope blocking. The P value was determined by Spearman's correlation.

We next evaluated whether immunization in guinea pigs that produced antibodies to multiple crucial gD2 epitopes offered added protection against genital lesions compared with immunization that produced antibodies to few crucial epitopes. An epitope was considered blocked if it showed any competition (≥1%) with the community prototype MAb for binding to gD2. We chose ≥1% as positive for blocking, because the biosensor value is generally a negative number when IgG fails to block gD2 binding. Animals were assigned the same numbers as in Figs 1–3. The number of separate epitopes blocked ranged from 2 to the maximum of 7 with a mean of 4.2 (Fig 6A). The genital disease scores in animals were arranged in the same order as Fig 6A now according to the number of epitopes blocked (Fig 6B). We detected a highly significant correlation between the number of epitopes blocked and cumulative lesion days (Fig 6D). A breakpoint appears at ≥4 epitopes. The 18 animals that produced antibodies that blocked ≥4 epitopes had genital lesions on a mean of 1.9 ± 3.4 days compared with 7.3 ± 5.2 days for the seven animals that blocked ≤3 epitopes (p = 0.006, by Student’s t-test).

We performed a sensitivity analysis in which we used ≥5% blocking as the cutoff to consider an epitope blocked rather than ≥1%. The correlation between number of epitopes blocked and cumulative lesion days remained highly significant (r = -0.52, p = 0.008). Therefore, we detected a significant correlation between the epitopes blocked and genital lesions when we used 1% or 5% as the cut-off for blocking.

We evaluated the cumulative percent of epitopes blocked as another approach to assess the correlation between epitopes blocked and cumulative lesion days. We determined the cumulative percent of epitopes blocked by calculating the sum of blocking by each prototype MAb. For example, guinea pig #2 had a cumulative disease score of 13 and cumulative percent blocking of 37% that was based on blocking the MC2 epitope by 7% and the 1D3 epitope by 30%, but not blocking any other epitope (Figs 3 and 6C). The correlation between cumulative lesion days and cumulative percent epitope blocking was r = -0.42 (p = 0.04) (Fig 6F), which was less robust than the correlation between cumulative lesion days and number of epitopes blocked (r = -0.57). The results indicate that the number of epitopes blocked is a stronger correlate of cumulative disease days than the cumulative percent of epitopes blocked.

In human vaccine trials, the number of subjects that develop disease (incidence of disease) is generally used as an endpoint rather than the number of days with lesions (a measure of disease severity) [9, 10]. Therefore, we extended our analysis to evaluate the guinea pig results based on the incidence of genital disease, defined as one or more lesion days. Animals with no genital lesions produced antibodies that blocked significantly more epitopes compared with animals that developed genital lesions (Fig 6E), which is consistent with results using the cumulative number of lesion days as the endpoint.

In addition, we evaluated the antibody responses to each crucial epitope to determine whether the lack of a response to a particular epitope was associated with significantly more lesion days. Two animals failed to produce antibodies that blocked the pink DL11/77S epitope. These two animals had significantly more lesion days than the 23 animals that produced antibodies to that epitope. The lack of response to no other crucial epitope was significantly associated with more lesion days, although animals that failed to produce antibodies that blocked the brown MC14 and red MC23 epitopes had more lesion days that approached statistical significance (Table 2). These results suggest that antibodies to the receptor binding epitopes, represented by DL11/77S and MC23, and to a cell-to-cell spread epitope, represented by MC14, are particularly important components of the multiple epitope response required for protection.

**Table 2:** Lesion days based on antibodies produced to individual epitopes*.

	Pink (DL11/77S)		Yellow (1D3)		Green (MC2)		Brown (DL6)		Brown (MC14)		Blue (MC5)		Red (MC23)	
	+	-	+	-	+	-	+	-	+	-	+	-	+	-
No. animals	23	2	7	17	8	17	18	7	3	21	24	1	22	3
Mean cumulative lesion days	2.9 ± 4.3	9.5 ± 4.9	2.6 ± 4.8	3.9 ± 4.8	3.5 ± 5.1	3.4 ± 4.5	2.3 ± 3.5	6.4 ± 5.9	0	4.0 ± 4.8	3.0 ± 4.2	13	2.7 ± 4.2	8.7 ± 5.1
P value	0.04		0.39		0.74		0.11		0.07		NA		0.06	

*Data derived from Figs 1B and 3C–3I; NA, not applicable; P values calculated by Mann-Whitney.

### Epitopes missing from the antibody response to HSV-2 gD2 immunization of human volunteers

Whitbeck et al reported the epitope-specific antibody responses of 29 human volunteers immunized with the GSK gD2 vaccine in the Herpevac Trial [10, 25]. The samples selected for evaluation were obtained one-month after the third (final) immunization and were approximately equally represented by subjects that had high (>8,061), intermediate (2,463–8,060) or low (<2,463) gD2 ELISA titers [25]. Most subjects produced antibodies to the epitopes recognized by the pink, blue, green and red communities; however, few or none produced antibodies to the yellow or brown communities represented by 1D3, DL6 and MC14 (Table 3). The gD2 vaccine provided no protection against genital HSV-2 infection in the Herpevac Trial; therefore, we were not able to assess epitope specific correlates of protection. An interesting analysis that we were able to perform was to compare the epitope-specific antibody responses in gD2 immunized humans and guinea pigs (Table 3). The gD2 vaccine used in the guinea pig study differed from the Herpevac Trial in that gD2 containing 306 amino acids was used in guinea pigs, while 281 amino acids was used in humans. In addition, CpG and alum were used as adjuvants in guinea pigs compared with MPL and alum in humans. Importantly, both vaccines included all the epitopes evaluated with our gD2 MAb panel [10, 26].

**Table 3:** Epitope-specific antibody responses in gD2 immunized guinea pigs and humans.

MAb	Community	% Protection by MAb in mice*	Guinea pigs (% Positive)^#^	Humans^§^ (% Positive) [25].
DL11/77S	Pink	80%	23/25 (92%)	25/29 (86%)
1D3	Yellow	80%	7/24 (29%)	0/29 (0%)
MC2	Green	80%	8/25 (32%)	22/29 (76%)
DL6	Brown	70%	18/25 (72%)	1/29 (3%)
MC14	Brown	50%	3/24 (13%)	1/29 (3%)
MC5	Blue	50%	24/25 (96%)	21/29 (72%)
MC23	Red	30%	22/25 (88%)	20/29 (69%)

* Data derived from Fig 5.

^#^ Guinea pigs were immunized with gD2(306t) containing 306 amino acids administered with CpG and alum. Data is derived from Fig 3.

^§^ Humans were immunized with gD2 containing 281 amino acids administered with MPL and alum. Antibody responses in guinea pigs and humans were evaluated using gD2(285t) that contains 285 gD2 amino acids [30]. All epitopes tested are contained within both gD2 immunogens.

Table 3 includes the epitope specific antibody responses in humans (results from [25]), and guinea pigs (results from Fig 3), and the protection provided by MAb passive transfer in mice (results from Fig 5). Yellow (1D3) and brown (DL6) community antibodies were detected in 0% and 3%, respectively in humans immunized with gD2. In contrast, 29% of guinea pigs produced antibodies that blocked gD2 binding to 1D3 and 72% produced antibodies that blocked binding to DL6 (Table 3). MAbs 1D3 and DL6 protected 80% and 70% respectively of mice against death. Few brown community antibodies (MC14) were detected in either guinea pigs (13%) or humans (3%), yet MC14 protected 50% of mice (Fig 5) and each animal that produced antibodies that blocked this epitope was totally protected against genital lesions in guinea pigs (Table 2).

Our guinea pig results suggest that blocking 4 or more epitopes protects against genital lesions. The mean number of epitopes blocked by gD2 immunization in guinea pigs was 4.2 ± 1.3 compared with 2.9 ± 1.4 in humans (p = 0.001, by Student’s t-test) (human data derived by re-analysis of [25]). Nineteen of 25 guinea pigs immunized with gD2 produced antibodies that blocked one or more of the DL6, 1D3 and MC14 epitopes, compared with 2/29 humans (p = 0.0001, Fisher’s Exact Test). These results suggest that some important gaps may exist in the antibody responses produced by the gD2 vaccine in humans, particularly to the DL6, 1D3 and MC14 linear epitopes, while antibody responses to the conformational epitopes were robust in humans and guinea pigs.

## Discussion

HSV-2 gD2 mediates virus receptor binding, activation of gH/gL and cell-to-cell spread, which are crucial viral functions [12, 37]. We hypothesized that an optimal gD2 vaccine is one that produces antibodies to multiple crucial epitopes involved in these functions. The high throughput biosensor technology provides a powerful new tool for measuring epitope-specific antibody responses to linear and conformational epitopes. We used the biosensor technology to demonstrate that some epitopes were immunogenic in almost all guinea pigs, while others were immunogenic in only a few. We determined in guinea pigs that the greater the number of crucial gD2 epitopes blocked by immunization, the better the protection was against genital lesions. We used MAb passive antibody studies in mice to demonstrate that MAbs to each of seven crucial gD2 epitopes provided significant, although partial protection against death and genital disease. Additional passive antibody studies in mice will be required to evaluate whether combinations of MAbs will provide maximum protection and to assess the extent of protection provided by the MAb IgG Fab domain compared with the Fc domain.

We demonstrated a strong correlation in guinea pigs between both neutralizing and ELISA titers with protection against genital lesions. In the Herpevac Trial, ELISA titers correlated with protection again HSV-1 genital infection [10, 29]. The geometric mean gD2 ELISA titer in the Herpevac Trial was 1:6809, which is not very different from the geometric mean ELISA titer of 1:8922 noted in our guinea pig studies [10]. In contrast, after the final immunization, the mean HSV-2 neutralizing titer in the Herpevac Trial for Women was 1:29, which is radically different from the geometric mean neutralizing titer of 1:893 in our guinea pig studies [10, 38]. In addition, in the Herpevac Trial, the mean neutralizing antibody titers fell to undetectable levels by study month 16 [10]. The strong correlation in guinea pigs between neutralizing antibodies and protection against genital lesions raises important concerns about the low neutralizing antibody response in the Herpevac Trial.

Some researchers have proposed an important role for ADCC in protecting vaccinated mice against HSV, although the target antigens for the ADCC antibodies have not be defined [33, 34, 39]. The single cycle live virus vaccine candidate in those studies lacks gD2, which is a major target of neutralizing antibodies; therefore, it is not surprising that alternate mechanisms of protection assume greater importance for that vaccine in murine models [40]. Our guinea pig results provide strong evidence that neutralizing antibodies contribute significantly to protection in gD2-immunized animals. Two other studies have also reported a correlation between neutralizing antibodies and protection against genital disease in gD2-immunized guinea pigs; however, a third report failed to detect this correlation [41–43]. In humans, studies of neonatal herpes have demonstrated that neutralizing antibodies in the newborn protect against the disseminated form of the disease [44, 45]. A human neonatal herpes study evaluated both neutralizing antibodies and ADCC titers in newborns and noted that both independently correlate with protection against neonatal HSV infection [46]. Similar findings were reported in mice [47]. In addition, passive transfer studies in adult mice demonstrated that protection against intravaginal infection was mediated by neutralization and ADCC [48]. Therefore, both neutralizing antibodies and ADCC contribute to protection, with one or the other assuming greater importance depending on the immunogen used and both contributing to protection during natural infection.

Neutralization in the absence of complement is mediated by the IgG Fab domain, while ADCC requires the IgG Fc domain. Our guinea pig results demonstrated a significant correlation between neutralizing titers and protection, indicating a role for the IgG Fab domain. Evidence to support a possible contribution of the IgG Fc domain to protection comes from some passive transfer experiments. MAb MC23 from the red community is an IgG1 MAb that blocks the nectin-1 receptor. It protected only 30% of mice against death despite being a potent neutralizing antibody with an endpoint titer of 0.26 μg/ml. DL11 from the pink community is an IgG2a MAb that blocks both the nectin-1 and HVEM receptors [37]. It has a similar neutralizing antibody titer as MC23 and protected 80% of mice, which was significantly better than MC23. MC23 binds only to FcγRIII, while DL11 binds to FcγRIII at a higher endpoint titer than MC23 and also binds to FcγRIV. These results support a possible contribution of ADCC to protection mediated by DL11. An alternate explanation, however, is that protection is mediated by the Fab domain based on DL11 binding to gD2 with 81-fold higher avidity (slower off rate in Table 1) than MC23.

Additional passive transfer studies will be required to assess the relative contributions of the Fab and Fc domains of antibodies targeting crucial gD2 epitopes in protecting animals against genital HSV-2. Approaches include passive transfer with MAb Fab domains, using antibodies to various murine Fc_γ_Rs to block their activity, and performing experiments in IgG Fc receptor knockout mice [35, 39]. To fully evaluate the contribution of the IgG Fc domain in antibody protection, experiments will require assessing the impact of HSV-1 and HSV-2 gE. These glycoproteins function as IgG Fc receptors that greatly diminish the ability of the IgG Fc domain to activate complement and mediate ADCC [32, 49, 50]. Greater protection may have been observed during our passive transfer studies if the gE2 Fc binding domains were non-functional. A goal of our gC2/gD2/gE2 trivalent subunit antigen vaccine is to produce antibodies that target crucial functional epitopes with their Fab domains while improving IgG Fc activity by generating antibodies that block the immune evasion properties of gE [26].

The comparison of epitope specific antibody responses in guinea pigs and humans is instructive. The gD2 vaccine protein that we used in guinea pigs has 306 amino acid residues and includes the 281 amino acids used in the gD2 human Herpevac trial [10]. The gD2 vaccine in the Herpevac trial failed to produce significant levels of antibodies that blocked gD2 binding to yellow (1D3) and brown (DL6 and MC14) epitopes [25]. The response in guinea pigs was more robust than in humans, yet many guinea pigs also failed to produce antibodies to these epitopes. The observation that passive transfer of MAbs to these epitopes partially protected mice against a lethal HSV-2 infection suggests that this deficiency in coverage is important. The 1D3, DL6 and MC14 epitopes are linear. Therefore, one approach to improve the immunogenicity of these epitopes is to systematically substitute amino acid sequences along the 8–11 amino acids that comprise the epitopes and then assess the immunogenicity of the mutated epitope. Another option to improve epitope immunogenicity is to administer peptides with adjuvants, a third option is to remove glycosylation sites and a fourth is to evaluate different adjuvants [41].

Many vaccine trials in animal models have used gD2 as an immunogen and numerous studies have assessed protection by antibody passive transfer in mice [27, 28, 41–43, 51–59]. Some animal and human studies have also evaluated epitope-specific antibody responses to gD2 immunization [25, 42]. Our report differs from prior studies in that we correlated antibody responses to crucial gD2 epitopes with protection against genital lesions. Measuring epitope specific antibody responses to crucial glycoprotein epitopes provides a novel approach to improve the accuracy of animal models in predicting outcomes in human trials. The disappointing results of prior human trials suggest that efforts at improving animal models are important. We propose that future phase I/II human immunogenicity trials that include gD2 as an immunogen assess whether high titers of neutralizing antibodies are produced and determine the breadth of the antibody response to an array of four or more crucial epitopes. Preferably, the antibodies produced will include one or more of the linear 1D3, DL6 and MC14 epitopes that were largely absent in the Herpevac Trial for women. Attempts to modify the immunogen or adjuvant are warranted prior to large phase III human trials if deficiencies are noted.

## Methods

### Ethics statement

Female Hartley strain guinea pigs and female BALB/c mice were obtained from Charles River Laboratories (Wilmington, MA). The animals were used for studies in accordance with protocol No. 805187 approved by The Institutional Animal Care and Use Committee of the University of Pennsylvania. The protocol followed recommendations in the Institute for Laboratory Animals Research’s “Guide for the Care and Use of Laboratory Animals.” Subcutaneous saline was given to animals that appeared dehydrated. Meloxicam was used to control pain once genital lesions developed. For euthanasia, Euthasol was administered to guinea pigs while CO2 was administered to mice according to the recommendations of the Panel on Euthanasia of the American Veterinary Medical Association.

### Guinea pig immunization and challenge

Sera were obtained from two prior experiments performed in gD2 immunized guinea pigs [26]. In that study, animals were bled two weeks after the third immunization and challenged intravaginally with 5x10^5^ PFU of HSV-2 strain MS. Days with genital lesions were recorded post infection for 60 days.

### Viruses, MAbs and proteins

HSV-2 strain MS was grown and titered on African green monkey kidney (Vero, ATCC CRL-1586) cells [60]. Baculovirus gD2(285t) and gD2(306t) were produced in insect sf9 cells and purified from the supernatant fluids [31, 60]. Glycoprotein D-specific MAbs have been previously described [30].

### IgG purification

IgG was purified from guinea pig sera taken two weeks after the final immunization using a Protein A Spin Plate for IgG according to the manufacturer's instructions (Thermo Scientific, Pierce Biotechnology, Rockford, IL). IgG-containing fractions were stored at −80°C. The protein concentration of purified IgG was determined using the Micro BCA Protein Reagent Kit (Thermo Scientific, Pierce Biotechnology, Rockford, IL).

### Neutralizing and ELISA antibody endpoint titers

Neutralizing antibody titers were measured in the absence of complement as described in our prior publication [26]. Endpoint ELISA titers were performed as previously described except serial two-fold dilutions of purified IgG were added to the plates instead of a 1:1000 dilution of serum [26]. Endpoint titers were calculated by regression analyses as the IgG dilution giving an OD reading 2-fold higher than background.

### Cell-to-cell spread assays

Human epidermal cells (HaCaT) (provided by H. Diggelmann, ISREC, Switzerland) were grown to confluence on 48-well tissue culture plates (Costar) [61]. Each well was inoculated with approximately 30 PFU of HSV-2 strain MS. Virus infection was allowed to proceed for 1 h at 37°C, then virus that had not entered cells was removed by citric acid wash at pH3.0 for 1 min at RT followed by a PBS wash. Serial 4-fold dilutions ranging from 40 μg/ml to 0.67 μg/ml of IgG purified from MAbs DL6, MC14, MC16, and from rabbit anti-gE2 polyclonal serum R265 were added to wells in 250 μl of DMEM containing 5% fetal bovine serum and incubated at 37°C in 5% CO_2_ for 48 h [32]. Monolayers were stained with crystal violet and plaque size measured using an inverted microscope fitted with an eyepiece micrometer [61].

### ADCC assays

Antibody-dependent cellular cytotoxicity (ADCC) endpoint titers were determined using murine FcγRIII and FcγRIV ADCC Reporter Bioassays according to the manufacturer’s instructions (Promega Corporation, Madison, Wisconsin). Briefly, white with clear, flat-bottomed 96-well plates (Falcon REF 353377, Corning Incorporated, Big Flats, NY) containing approximately 1.2X10^4^ Vero cells/well were infected with HSV-2 strain MS at an MOI of 2 for 16h. At the time of the assay, 95% of the culture medium was removed from each well and replaced with 25μl of RPMI 1640 supplemented with 4% low IgG serum (RPMI low IgG serum) and 25 μl purified gD2-specific MAb or control antibodies that had been serially diluted 5-fold in RPMI low IgG serum, and incubated at 37°C in 5% CO_2_ for 30 min. Jurkat cells stably expressing murine FcγRIII or FcγRIV were added as effector cells at an effector to target ratio of 6:1 in a volume of 25 μl and incubated at 37°C in 5% CO_2_ for 6 h. 75 μl of Bio-Glo Reagent was then added to each well, and luminescence was determined using a Luminoskan Ascent Luminometer (Thermo Scientific). Fold-induction was calculated using the following equation: Fold induction = RLU (induced—background) / RLU (no antibody control—background). Endpoint titers were calculated as the IgG dilution yielding a ≥2-fold induction.

### Mouse passive transfer and challenge

Seven- to eight-week old female BALB/c mice (Charles River Laboratories, Wilmington, MA) were treated subcutaneously with 2 mg of medroxyprogesterone 5 days prior to challenge to induce the diestrus stage of the estrus cycle that increases susceptibility to HSV-2 intravaginal infection [62]. One day prior to challenge, mice received a passive transfer of 100 μg of MAb IgG/mouse intraperitoneally (IP). On the day of challenge, the vagina of each mouse was cleared using a sterile cotton swab moistened with PBS, followed by an intravaginal challenge with HSV-2 MS (5X10^3^ PFU/mouse, approximately 500 LD_50_). The virus used for inoculation was titered to ensure accurate dosage (5X10^3^ PFU/mouse). Mice were followed for survival for 10 days post-infection, weighed daily and scored for genital disease on a scale of 0–4 by assigning one point each for erythema, exudate, hair loss and necrosis of genital tissues [32].

### High throughput biosensor-based antibody competition assay

Antibody competitions were performed at RT using the Carterra Microfluidics continuous flow microspotter surface plasmon resonance imaging (CFM/SPRi) system [30]. Briefly, a panel of distinct MAbs recognizing gD were amine-coupled to a CDM200M sensor chip (Xantec GmbH) in a 96-spot format. The sensor chip was then moved to the SPR imager (IBIS MX96), blocked with ethanolamine, and primed with a running buffer of PBS-0.01% Tween 20. Antibody competitions were performed in a pre-mix assay format by saturating soluble gD2(285t) at a concentration of 75 ng/reaction with purified guinea pig IgG at 2 μg/reaction in 200 μl total volume. Each gD2(285t):guinea pig IgG mix was flowed across the sensor chip spotted with the gD MAbs. After each gD2(285t):guinea pig IgG mix, the chip surface was regenerated using 10 mM glycine pH 2.0 prior to flowing the next gD2:guinea pig IgG. The starting and ending (post-regeneration) response unit (RU) value of zero for each MAb spot indicates that both the guinea pig IgG and the gD2 were removed. Every 10th cycle gD2-only (without IgG) was flowed across the chip. The RU value obtained was used to establish the background binding of gD2 to compare with gD2 plus guinea pig IgG. Binding of gD2 establishes that the MAb is still present on the chip and has not lost its capacity to bind gD2 during regeneration. The blocking activity of the guinea pig IgG was calculated for each MAb as a percentage using the formula: [1-(RU gD2(285t) + GP IgG)/(RU gD(285t) alone)]*100. Purified gD2(285t) was used to screen for antibodies although gD2(306t) was used to immunize the guinea pigs. The rationale was based on MAbs in the pink community binding better to gD2(285t) than to gD2(306t), thus making the evaluation more reliable on gD2(285t) [30]. In addition, the samples from gD2 immunized humans were evaluated using gD2(285t), which facilitates comparisons between human and guinea pig responses [25].
